# Comparing the Metabolic Profiles Associated with Fitness Status between Insulin-Sensitive and Insulin-Resistant Non-Obese Individuals

**DOI:** 10.3390/ijerph191912169

**Published:** 2022-09-26

**Authors:** Shamma AlMuraikhy, Najeha Anwardeen, Aisha Naeem, Maha Sellami, Alexander Domling, Abdelali Agouni, Mohamed A. Elrayess

**Affiliations:** 1 Biomedical Research Center, Qatar University, Doha P.O. Box 2713, Qatar; 2 Groningen Research Institute of Pharmacy, Drug Design, Groningen University, 9712 CP Groningen, The Netherlands; 3 Ministry of Public Health, Doha P.O. Box 42, Qatar; 4 Department of Oncology and Pathology, Lombardi Comprehensive Cancer Center, Georgetown University Medical Center, 3800 Reservoir Rd., NW, Washington, DC 20007, USA; 5 Physical Education Department (PE), College of Education, Qatar University, Doha P.O. Box 2713, Qatar; 6 College of Pharmacy, QU Health, Qatar University, Doha P.O. Box 2713, Qatar

**Keywords:** physical activity, insulin sensitive, insulin resistant, non-obese

## Abstract

(1) Background: Young non-obese insulin-resistant (IR) individuals could be at risk of developing metabolic diseases including type 2 diabetes mellitus. The protective effect of physical activity in this apparently healthy group is expected but not well characterized. In this study, clinically relevant metabolic profiles were determined and compared among active and sedentary insulin-sensitive (IS) and IR young non-obese individuals. (2) Methods: Data obtained from Qatar Biobank for 2110 young (20–30 years old) non-obese (BMI ≤ 30) healthy participants were divided into four groups, insulin-sensitive active (ISA, 30.7%), insulin-sensitive sedentary (ISS, 21.4%), insulin-resistant active (IRA, 20%), and insulin-resistant sedentary (IRS, 23.3%), using the homeostatic model assessment of insulin resistance (HOMA-IR) and physical activity questionnaires. The effect of physical activity on 66 clinically relevant biochemical tests was compared among the four groups using linear models. (3) Results: Overall, non-obese IR participants had significantly (*p* ≤ 0.001) worse vital signs, blood sugar profiles, inflammatory markers, liver function, lipid profiles, and vitamin D levels than their IS counterparts. Physical activity was positively associated with left handgrip (*p* ≤ 0.01) and levels of creatine kinase (*p* ≤ 0.001) and creatine kinase-2 (*p* ≤ 0.001) in both IS and IR subjects. Furthermore, physical activity was positively associated with levels of creatinine (*p* ≤ 0.01) and total vitamin D (*p* = 0.006) in the IR group and AST (*p* = 0.001), folate (*p* = 0.001), and hematocrit (*p* = 0.007) in the IS group. Conversely, physical inactivity was negatively associated with the white blood cell count (*p* = 0.001) and an absolute number of lymphocytes (*p* = 0.003) in the IR subjects and with triglycerides (*p* = 0.005) and GGT-2 (*p* ≤ 0.001) in the IS counterparts. (4) Conclusions: An independent effect of moderate physical activity was observed in non-obese apparently healthy individuals a with different HOMA-IR index. The effect was marked by an improved health profile including higher vitamin D and lower inflammatory markers in IRA compared to IRS, and a higher oxygen carrying capacity and lipid profile in ISA compared to the ISS counterparts.

## 1. Introduction

According to the International Diabetes Association, there is a rise in diabetes all around the world, accounting for 425 million cases among individuals aged 20–79 years [1]. Evidence shows that by 2045, this number will rise to 629 million. The most common risk factors that increase the prevalence of diabetes are an unhealthy diet, sedentary lifestyle, and urbanization [1]. The state of Qatar has experienced an epidemiological change as its economy shifted from a healthy and active way of life (seafood diet, moving Bedouins, and pearl hunting) into sedentary office jobs during the last 5 decades, as it has become mostly dependent on gas and oil [2,3]. With a higher predisposition in women, the prevalence of type 2 diabetes mellitus (T2DM) among adult Qataris is strikingly high (16.7%) [4].

Despite the established correlation between obesity and insulin resistance, multiple studies have indicated that apparently healthy non-obese subjects could also develop insulin resistance and T2DM. They are classified as prediabetes as they manifest a high level of glucose, but lower than the diabetes threshold [5,6]. A recent study has identified that over 40% of young (<30 years old) lean/overweight females in Qatar are insulin resistant (IR) [4], compared to 25% of overweight females of other ethnicities [7,8,9]. Previous reports have indicated that insulin resistance could predict up to 80% of T2DM cases in non-obese subject individuals [9], suggesting that over 30% of overweight Qatari individuals are prone to T2DM [10].

Physical inactivity is an important risk factor for metabolic diseases, including insulin resistance, T2DM, and cardiovascular disease [5,11]. However, the underlying mechanisms through which physical activity protects against the different components of metabolic diseases remain not fully understood [12]. Regular physical exercise has a profound impact on human blood sugar regulation and insulin sensitivity [13]. Indeed, the adoption of a healthy lifestyle can change the life of a diabetic patient [11,14,15]. Studies have shown that 5 years of controlled diet and exercise reduce the risk of T2DM in non-obese individuals with glucose intolerance [12]. Recent studies in rats [16,17] and humans [18] showed that the skeletal muscle sensitivity to insulin increased after acute exercise and physical training. Other studies have also shown that glucose tolerance and insulin sensitivity, as well as various lipid parameters, blood pressure, and fibrinolytic activity, could be improved by regular exercise in IR individuals [18,19,20].

Studies comparing the effect of exercise in healthy non-obese insulin-sensitive and IR individuals remain limited. Understanding the underlying etiology of insulin sensitivity in this apparently healthy non-obese group would help in preventing the development of metabolic diseases including T2DM and in controlling disease progression through targeted diet and exercise [21,22,23]. In this study, the effect of physical activity on various clinically relevant metabolic traits was determined in apparently healthy non-obese Qatari individuals stratified by insulin sensitivity using data from the Qatar Biobank.

## 2. Materials and Methods

### 2.1. Data Source and Study Participants

**Figure 1:**
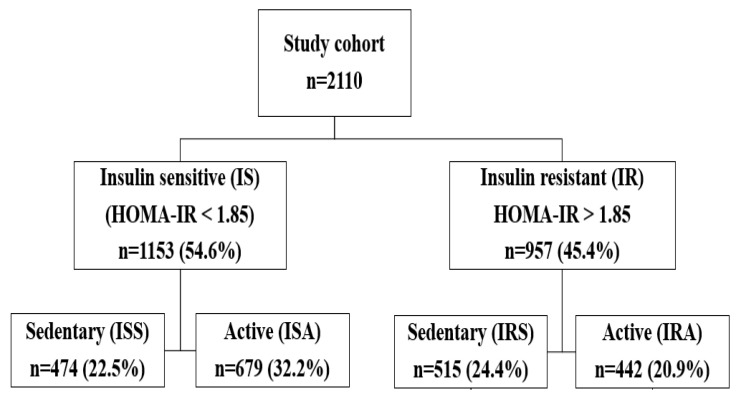
Study design indicating the study groups (ISA, ISS, IRA, and IRS) and their percentages.

This is a retrospective study. The data from 2110 participants was extracted from the Qatar Biobank, including questionnaires related to physical activity and laboratory results for 66 clinically relevant metabolic traits, such as measurements of systolic and diastolic blood pressure, waist-to-hip ratio (WHR), body mass index (BMI), clinical chemistry, and endocrinology tests (Table 1). The study was approved by the Institutional Review Boards of the Qatar Biobank (QF-QBB-RES-ACC-00066) and the Qatar University (QU-IRB 1716-E/22). All participants provided informed consent. Insulin resistance was determined by HOMA-IR ((fasting glucose (mmol/L) × fasting insulin mlU/mL)/22.5)). Individuals with an HOMA-IR less than or equal to 1.85 were categorized as IS, whereas those with an HOMA-IR greater than 1.85 were categorized as IR. Physically active participants were identified as those who walk at least two days per week for more than 150 min [24,25,26,27]. Inclusion criteria included young (20–30 years old), lean/overweight (BMI: 20–30 kg/m^2^), healthy (no chronic diseases) individuals. Exclusion criteria included those younger than 20 years or over 30 years old, with BMIs less than 20 kg/m^2^ or greater than 30 kg/m^2^, or participants with the following chronic diseases: diabetes, glaucoma, macular degeneration, blood clots, cardiovascular disease, bariatric surgery, or cancer. Among all participants, 51.5% were males and 54.6% were IS, including 22.5% sedentary (ISS) and 32.2% active (ISA), whereas 45.4% were IR, including 24.4% sedentary (IRS) and 20.9% active (IRA) (Figure 1).

### 2.2. Statistical Analysis

All analyses were performed using R Studio (v 4.0.3) software (R foundation for statistical computing, Vienna, Austria). Nominal variables are displayed as numbers with percentages, and the differences were determined using the chi-squared test. Numeric (continuous) data are presented as means (standard deviation) and were compared using Student’s t-test/Mann–Whitney U test. The Shapiro–Wilk test was used to examine the normality of the distribution and the skewed data were log-transformed. Differences in the marginal means of each metabolic profile (as the y variable) between active and sedentary at each level of HOMA-IR were calculated using the R emmeans package while correcting for confounders’ age, BMI, gender, and fasting time. Nominal *p*-values were corrected for multiple testing using the false discovery rate (FDR) method. The analysis incorporated the interaction between the HOMA-IR category and the physical activity groups; the estimate represents the offset in the intercept from the sedentary baseline to active individuals.

## 3. Results

### 3.1. Differences between IS and IR Individuals Regardless of Physical Activity Status

#### 3.1.1. BMI, Pulse Rate, Glucose, and C-Peptide Levels

As shown in Table 1, IR participants had significantly worse vital signs, including a higher BMI, systolic and diastolic blood pressure (BP), and pulse rate, than their IS counterparts. IR individuals also exhibited worse blood sugar profiles (fasting blood glucose, HBA1C, and C-Peptide).

#### 3.1.2. Hematological Parameters, Liver Function Tests, and Lipid Profile

Individuals in the IR group exhibited higher inflammatory markers (white blood cell count, neutrophil percentage, and absolute count). Similarly, liver function tests (uric acid, total protein, homocysteine, alkaline phosphatase (ALP), alanine aminotransferase (ALT), aspartate aminotransferase (AST), and gamma glutamyl transferase-2 (GGT-2)) and lipid profiles (triglycerides) showed higher levels in IR group.

#### 3.1.3. Iron Profile and Hormones

Higher free triiodothyronine, total iron-binding capacity (TIBC), unsaturated iron-binding capacity (UIBC), and ferritin levels were measured in the IR group. On the other hand, IR individuals exhibited lower levels of urea, homocysteine, creatine kinase, creatine kinase-2, total bilirubin, high density lipoprotein (HDL) cholesterol, testosterone, estradiol, sex hormone-binding globulin (SHBG), iron, folate, dihydroxyvitamin D, and vitamin B12. 

### 3.2. Comparing the Effect of Physical Activity in IS and IR Subjects

When assessing the impact of physical activity, active IS and IR (ISA and IRA) participants showed lower pulse rates, WBC, neutrophils, and lymphocyte counts, but higher weight, handgrip, red blood cell count, urea, uric acid, creatinine, CK, CK2, AST, and dihydroxyvitamin D levels than their sedentary counterparts. When comparing ISA versus ISS, ISA had higher levels of systolic BP, waist size, waist-to-hip ratio, hemoglobin, hematocrit, monocyte %, homocysteine, total bilirubin, albumin, ALT, total testosterone, iron, UIBC, ferritin, and folate levels, but lower C-peptide and estradiol than the ISS counterparts. When comparing IRA versus IRS, IRA showed lower C-reactive protein levels, fasting time, and glucose than the IRS counterparts (Table 1).

### 3.3. Differential Metabolic Response to Physical Activity in IS vs. IR

**Figure 2:**
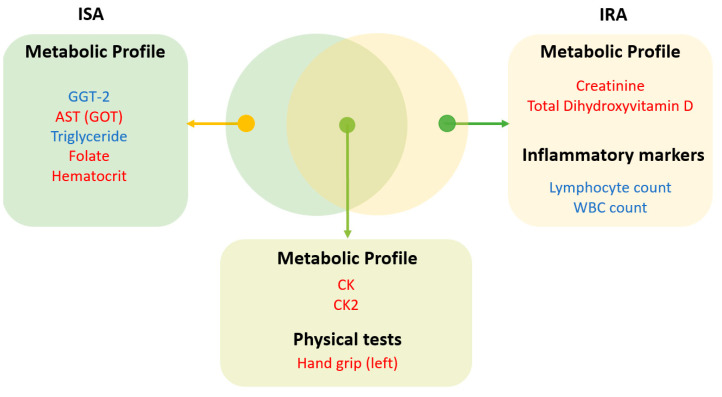
Venn diagram representing the results from the linear regression analysis, incorporating the interaction between the HOMA-IR category and physical activity groups (visual representation of Table 2). Red/blue shows the clinical measurements that were higher/lower in the physically active group, respectively.

To evaluate the association between physical activity and metabolic profiles in IS versus IR individuals, linear regression was conducted after adjusting for age, BMI, gender, and fasting blood glucose. As shown in Table 2, physical activity was positively associated with left handgrip, creatine kinase, and creatine kinase-2 in both IS and IR subjects. Furthermore, physical activity was positively associated with AST, folate, hematocrit, and estimated maximum heart rate in the IS groups only, whereas it was associated with creatinine, total dihydroxyvitamin D, and weight in the IR group. Conversely, physical inactivity was negatively associated with triglycerides and GGT-2 in the IS individuals, whereas it was negatively associated with white blood cells and an absolute number of lymphocytes in the IR subjects. Data is also summarized in Figure 2.

This analysis incorporated the interaction between the HOMA-IR category and physical activity groups; the estimate represents the offset in the intercept from the active individuals to sedentary baseline.

## 4. Discussion

The association between obesity and insulin resistance is well established, however, studies investigating the increased risk of insulin resistance in non-obese apparently healthy individuals and the effect of exercise on mitigating their risk remain limited. In this study, we focused on investigating the effect of physical activity on circulating levels of clinically relevant metabolic traits associated with insulin resistance in healthy lean and overweight (BMI: 20–30 kg/m^2^) individuals. Our data has shown that physical activity can exert an independent effect in non-obese apparently healthy IS and IR groups as it improves some, but not all traits associated with the risk of metabolic syndromes depending on HOMA-IR status. The emerging data can help assess the impact of physical activity in apparently healthy but high-risk subjects for a better understanding of exercise’s protective effects and a better management of the subjects’ risk.

### 4.1. Association between IR and BMI, Pulse Rate, and Hematological Parameters

As expected, our data revealed that overall IR individuals exhibited worse vital signs and a higher risk of diabetes, inflammation, liver disease, and hypertriglyceridemia than their IS counterparts. Our results showed significant elevations in WBC count and neutrophil percentages in IR individuals but decreased lymphocyte percentages. The WBC count is commonly used to monitor increased risks of insulin resistance and cardiovascular diseases in T2D patients as well as healthy individuals [28,29]. Neutrophils are the first immune cells to respond to inflammation and can exacerbate the chronic inflammatory state [30]. The neutrophil–lymphocyte ratio (NLR) is used as an indicator of subclinical inflammation. Increased NLR was significantly associated with IR, and high NLR values may be a reliable predictive marker of insulin resistance [31]. Our data also indicated an association between insulin resistance and RBC count. Our findings provide in vivo evidence of a relation between hyperinsulinaemia/insulin resistance, the main variables of insulin resistance syndrome and erythropoiesis. Increased red blood cell count could be considered a new aspect of the insulin resistance syndrome that could contribute to the increased risk of developing cardiovascular problems [32].

### 4.2. Association between IR and Kidney and Liver Function Tests

Our results also indicated higher levels of uric acid and total protein in IR individuals compared to their IS counterparts. Uric acid represents a reliable biomarker of IR subjects with metabolic syndromes. Elevated levels of uric acid are caused by various hemodynamic abnormalities, including glucose intolerance, insulin resistance, dyslipidemia, and hypertension. High uric acid levels can initiate oxidative stress, inflammation, and enzymes associated with glucose and lipid metabolism, suggesting a mechanism for the impairment of metabolic homeostasis [33]. Our results also showed lower levels of total bilirubin and albumin in IR compared to IS, but higher levels of ALP, ALT, and GGT2. These results are in line with a previous study that showed the association between lower levels of serum total bilirubin with an increased risk of T2D [34]. High levels of bilirubin are also negatively associated with abdominal obesity, hypertriglyceridemia, and cardiometabolic risk factors, including dyslipidemia and hypertension [35,36]. Conversely, increased ALP activity is correlated with various cardiometabolic diseases. Serum ALP levels are independently and positively associated with the surrogate markers of insulin resistance (triglyceride-to-high-density lipoprotein cholesterol ratio, triglyceride, and glucose) in the general population [37]. Additionally, insulin resistance was significantly associated with elevated ALT, AST, and GGT levels in nondiabetic adults, especially among those who were overweight/obese [38]. 

### 4.3. Association between IR and Hormones and Lipid Profile

As expected, our results showed significantly higher triglycerides and lower HDL cholesterol levels in IR individuals compared to their IS counterparts. This is in line with previous studies showing higher serum levels of triglycerides and low levels of HDL cholesterol in patients with metabolic syndrome [4,39,40,41,42,43]. Studies have also shown that the triglyceride/HDL ratio is positively associated with ALT levels and that assessment of IR and metabolic syndrome can become more precise by evaluating the TG/HDL cholesterol ratio and ALT, simultaneously [44,45]. Our data also indicated lower levels of testosterone and SHBG in the IR group. Testosterone levels are partly influenced by insulin resistance [46]. Testosterone deficiency is common in men with diabetes, regardless of the type. Low SHBG levels are correlated with low free testosterone even after HOMA-IR adjustment, suggesting that SHBG can be associated with testosterone deficiency beyond the influence of insulin resistance [47]. Our results also showed significantly higher free triiodothyronine (T3) in IR compared to IS. High levels of free T3 are associated with insulin resistance, and the use of free T3 to assess insulin resistance in healthy patients was previously suggested [48]. Indeed, previous studies have shown that higher baseline free T3 levels were significant predictors of decreased insulin sensitivity [49]. 

### 4.4. Association between IR and Iron Profile and Vitamins

Our results also showed significantly lower iron levels in IR which correlated significantly with higher TIBC and UIBC results. UIBC is used along with a serum iron test and TIBC to evaluate iron deficiency or iron overload, where a high TIBC usually indicates iron deficiency anemia [50]. Furthermore, our results showed lower levels of dihydroxyvitamin D in the IR group. Vitamin D deficiency can play a role in the development of insulin resistance in individuals with prediabetes who have a high cardiovascular risk [51]. Recent studies reported inverse correlations between the vitamin D status, hyperglycemia, and glycemic control in patients with T2D [52]. Additionally, IR had significantly lower levels of vitamin B12 and folate compared to the IS counterpart. Low B12 levels in pregnancy alter adipose-derived circulating micro RNAs, which may mediate an adipogenic and IR phenotype, leading to obesity [53]. A recent study showed that poor folic acid, vitamin D, and B12 status were associated with insulin resistance in nondiabetic obese patients [54]. Early supplementation of folate and vitamin B12 improved insulin resistance and lipid levels in intrauterine growth-retardation rats to some extent, along with decreasing homocysteine levels, but not enough to completely repair glucose and lipid metabolism [55].

Physical activity can induce adaptations in metabolism that are considered beneficial for health. Intense and continuous exercise, training, and competitions were reported to induce changes in the serum concentrations of numerous laboratory parameters [56]. Regular mild exercise, especially aerobic training, has been widely shown to improve cardiovascular and pulmonary function [25]. Furthermore, it affects the lipid profile and the risk of cardiovascular diseases. Our emerging data showed changes in the response to mild activity in various clinically relevant traits common between apparently healthy non-obese ISA and IRA groups, in addition to other traits specific to ISA or IRA.

### 4.5. Association between Physical Activity and Cardiac Markers

Among the common clinically relevant traits that were associated with physical activity regardless of insulin resistance status, our results showed elevated levels of both CK and CK2 in addition to the expected higher handgrip. CK is an enzyme found in the brain (CK1), skeletal, and heart muscles (CK2 and CK3). Normal levels of CK in the blood are mainly derived from skeletal muscles. Damaged tissues cause higher CK levels in the blood [57,58]. CK and CK2 increase with exercise, regardless of insulin resistance status, since they mark muscle damage associated with greater physical activity. Among the clinically relevant traits that are associated with ISA, but not IRA, our results showed higher levels of AST. Elevated AST release is typical of exercising muscle [56]. In our data, the elevation in AST in ISA, but not IRA, could simply reflect the lower AST levels in the ISS compared to IRS, suggesting a lower risk of liver disease in the former group. Our results also showed lower GGT2 levels in ISA, but not IRA. GGT is an extracellular membrane-bound enzyme that cleaves the peptide bonds of gamma-glutamyl in glutathione and transfers the gamma-glutamyl moiety to acceptors. Studies have shown that levels of GGT2 are higher in individuals with liver disease, and physical activity can lower these levels [59]. Elevated GGT2 seen in all IR could indicate a risk of liver disease. The effect of exercise on lowering GGT2 levels in IS, but not IR, could also indicate could also indicate lowering risk of liver disease.

### 4.6. Association between Physical Activity and Hematological Parameters

Our results also showed higher levels of folate in ISA, but not IRA, suggesting that exercise improved folate levels in IS but not IR individuals, although a similar trend was seen in the IRA group. Folate supplementation increases insulin sensitivity [55]. The modest increase in folate levels in ISA could reflect supplement intake, although, the interaction between exercise and folate levels requires further investigation. Our data also showed that ISA individuals exhibit significantly higher hematocrit concentration. Improvement in hematocrit concentration during exercise is known [60]. Exercise can increase the total hemoglobin, red blood cell mass, and hematocrit, which increases oxygen carrying capacity. During exercise, the body’s demand for oxygen is increased to provide muscles with the required oxygen for energy production [61,62]. Indeed, athletes have a higher total mass of red blood cells, hemoglobin, and hematocrit compared to sedentary subjects [61]. The increase in hematocrit in ISA, but not IRA, could suggest a better oxygen carrying capacity in the former than the latter since higher oxygen uptake was previously described as an important determinant for insulin sensitivity [63]. Our data showed an inverse relationship between physical activity and WBC and absolute lymphocyte count in IRA individuals, confirming the anti-inflammatory role of exercise, especially in the IR group, since IRS showed a higher WBC count and absolute lymphocyte. Previous reports showed that exercise reduces WBC counts and that changes in the total WBC are inversely correlated with changes in the glucose disposal rate [64]. Our data also revealed that other clinically relevant traits were associated with IRA, but not ISA. These include elevated creatinine levels, although a similar trend of increase was seen in the IS group. Creatinine levels are elevated after exercise as it plays a role in providing energy to muscle tissues [65]. Various studies have shown that athletes exhibit elevated creatinine levels [66,67]. Creatinine is a metabolic product of creatine phosphate in muscles, which provides energy to muscle tissues. The elevated creatinine levels in IRA, but not ISA, may reflect greater muscle damage in the IRA group.

### 4.7. Association between Physical Activity and Lipid Profile and Vitamin D

Triglyceride levels were slightly, but significantly, lower in the ISA group. The interaction of insulin sensitivity and triglyceride levels with exercise is well established. Previous studies have shown that increased physical activity improves insulin sensitivity and triglyceride levels [68]. The lack of effect of physical activity on triglyceride levels in the IR group requires further investigation. Another metabolite that increased in IRA was the 1,25-dihydroxyvitamin D. Previous studies have shown that serum 1,25-dihydroxyvitamin D levels increase with exercise [69]. Our data showed lower levels of vitamin D in IRS than ISS, which may explain the higher response to exercise in the IRA group, perhaps due to a greater exposure to sunlight [70]. 

### 4.8. Difference in the Metabolic Profile Due to Physical Activity between Males and Females of Different HOMA-IR Groups

We found a significant increase in creatine kinase, creatine kinase 2, folate, bilirubin, and left handgrip in physically active males, whereas a decrease in waist size, triglyceride, and GGT2 levels was calculated. On the other hand, a similar analysis in females showed significant changes in only creatinine levels due to activity, and no significant associations between other metabolites due to physical activity were found (Tables S1 and S2).

This study has potential limitations including the lack of osmolarity data. Indeed, research has shown that potential hyperglycemic conditions might affect fluid balance and hydration status, especially during physical activity, and this might have a significant effect on the level of blood biomarkers and on metabolic and vascular health profiles. Furthermore, fluid restriction has been reported to affect a cascade of health biomarkers due to raised copeptin levels, particularly in diabetic patients [71,72,73]. 

## 5. Conclusions

This study has revealed an independent effect of physical activity in non-obese apparently healthy IS and IR individuals, marked by higher vitamin D and lower inflammatory status in the IRA compared to the IRS group and a higher oxygen carrying capacity and lipid profile in ISA compared to the ISS counterparts. The emerging data could help in understanding the protective role of moderate physical activity for the improved management of non-obese apparently healthy subjects with a higher risk of insulin resistance and T2D.

## Figures and Tables

**Table 1:** General characteristics of participants.

		Insulin Sensitive			Insulin Resistant			*p* Value			
		All (1153)	Sedentary (474)	Active (679)	All (957)	Sedentary (515)	Active (442)	ISS vs. ISA	IRS vs. IRA	IS vs. IR	ANOVA
**Vital signs**	Age	25.9 (2.9)	25.8 (3.0)	26.0 (2.8)	25.7 (3.1)	25.8 (3.1)	25.6 (3.1)	0.327	0.349	0.186	0.194
	BMI (continuous)	24.5 (2.6)	24.3 (2.6)	24.7 (2.6)	25.6 (2.7)	25.6 (2.7)	25.7 (2.6)	0.024	0.302	≤0.001	≤0.001
	Average systolic BP	105.2 (9.4)	103.7 (9.5)	106.2 (9.3)	108.4 (9.9)	107.8 (9.6)	109.0 (10.3)	≤0.001	0.065	≤0.001	≤0.001
	Average diastolic BP	62.3 (8.1)	62.6 (8.3)	62.1 (7.9)	64.6 (8.4)	65.0 (8.3)	64.0 (8.4)	0.387	0.062	≤0.001	≤0.001
	Average pulse rate	69.2 (10.4)	70.4 (9.8)	68.3 (10.7)	72.7 (10.4)	73.3 (10.2)	72.0 (10.7)	≤0.001	0.035	≤0.001	≤0.001
**Blood sugar marker**	Fasting Time (min)	539.3 (289.9)	530.1 (303.9)	545.8 (279.6)	458.7 (304.2)	439.3 (309.3)	481.1 (296.9)	0.165	0.019	≤0.001	≤0.001
	HOMA-IR	1.2 (0.4)	1.3 (0.3)	1.2 (0.4)	3.6 (3)	3.7 (3.0)	3.6 (3.0)	0.126	0.964	≤0.001	≤0.001
	C-Peptide (ng/mL)	4.7 (0.4)	1.5 (0.5)	1.4 (0.4)	4.9 (1.3)	2.8 (1.4)	2.7 (1.1)	0.020	0.969	0.524	≤0.001
	Insulin (uU/mL)	6.1 (1.7)	6.2 (1.7)	6.0 (1.7)	16.4 (11.3)	16.7 (11.8)	16.1 (10.7)	0.106	0.827	≤0.001	≤0.001
	HBA_1_C	5.1 (0.3)	5.1 (0.3)	5.1 (0.3)	5.2 (0.4)	5.2 (0.4)	5.2 (0.4)	0.535	0.884	≤0.001	≤0.001
	Glucose (mmol/L)	4.6 (0.4)	4.6 (0.4)	4.6 (0.4)	4.9 (0.8)	5.0 (0.9)	4.9 (0.6)	0.770	0.016	≤0.001	≤0.001
**Physical tests**	Sitting height	90.6 (13.7)	90.8 (15.1)	90.4 (12.7)	90.3 (12.7)	90.3 (13.2)	90.3 (12.0)	0.001	0.096	0.176	0.983
	Weight	68.1 (11.1)	66.2 (10.9)	69.5 (11.1)	71.7 (11.3)	70.6 (11.2)	73.0 (11.4)	≤0.001	0.002	≤0.001	≤0.001
	Waist size (cm)	77.1 (8.4)	76.1 (8.6)	77.8 (8.2)	80.7 (9.1)	80.2 (9.0)	81.2 (9.2)	≤0.001	0.084	≤0.001	≤0.001
	Hips size (cm)	99.6 (6.7)	99.6 (6.7)	99.6 (6.7)	101.7 (6.3)	101.5 (6.5)	101.9 (6.2)	0.954	0.395	≤0.001	≤0.001
	Waist-to-hip ratio	0.8 (0.1)	0.8 (0.1)	0.8 (0.1)	0.8 (0.1)	0.8 (0.1)	0.8 (0.1)	≤0.001	0.251	≤0.001	≤0.001
	Hand grip (left)	33.3 (13)	30.2 (12.1)	35.4 (13.2)	33.4 (12.9)	31.7 (12.6)	35.3 (13.1)	≤0.001	≤0.001	0.76	≤0.001
	Hand grip (right)	34.5 (13.1)	31.5 (12.5)	36.5 (13.1)	34.3 (12.8)	32.9 (12.7)	35.9 (12.8)	≤0.001	≤0.001	0.902	≤0.001
	Planned run time (s)	592.1 (160.5)	575.4 (174.0)	603.8 (149.3)	602.2 (155.7)	590.5 (167.8)	615.8 (139.4)	0.242	0.334	0.129	≤0.001
**Blood inflammatory markers**	Hemoglobin (g/dL)	13.9 (1.7)	13.5 (1.7)	14.1 (1.6)	13.9 (1.8)	13.8 (1.8)	14.0 (1.8)	≤0.001	0.076	0.383	≤0.001
	Hematocrit	41.2 (4.5)	40.2 (4.5)	41.9 (4.3)	41.4 (4.8)	41.2 (4.7)	41.7 (4.9)	≤0.001	0.070	0.272	≤0.001
	Red Blood Cell (×10^6^ μL)	5 (0.6)	4.9 (0.6)	5.0 (0.5)	5.1 (0.6)	5.1 (0.6)	5.1 (0.6)	≤0.001	0.013	≤0.001	≤0.001
	White Blood Cell (×10^3^ μL)	6.3 (1.8)	6.4 (1.9)	6.1 (1.7)	6.7 (1.8)	6.9 (1.9)	6.5 (1.7)	0.009	0.001	≤0.001	≤0.001
	Monocyte%	7.7 (2)	7.5 (1.9)	7.8 (2.0)	7.7 (2.1)	7.7 (2.0)	7.8 (2.2)	0.020	0.540	0.546	0.166
	Neutrophil (×10^3^ μL)	3.4 (1.4)	3.5 (1.4)	3.3 (1.4)	3.7 (1.4)	3.8 (1.5)	3.5 (1.3)	0.011	0.003	≤ 0.001	≤0.001
	Neutrophil%	52.1 (10.4)	52.3 (10.8)	51.9 (10.0)	53.9 (9.9)	54.2 (10.1)	53.5 (9.7)	0.166	0.228	≤0.001	≤0.001
	Lymphocyte (×10^3^ μL)	2.2 (0.6)	2.3 (0.7)	2.2 (0.6)	2.3 (0.7)	2.3 (0.8)	2.2 (0.6)	0.010	0.008	0.054	0.002
	Lymphocyte%	36.9 (9.1)	36.7 (9.4)	36.9 (9.0)	35 (8.9)	34.9 (9.1)	35.2 (8.7)	0.398	0.395	≤0.001	≤0.001
	Eosinophil%	2.7 (2.1)	2.7 (2.0)	2.8 (2.1)	2.8 (2)	2.7 (1.7)	2.9 (2.3)	0.208	0.557	0.215	0.319
	Basophil%	0.6 (0.3)	0.6 (0.4)	0.6 (0.3)	0.6 (0.3)	0.6 (0.4)	0.6 (0.3)	0.647	0.435	0.891	0.844
	C-Reactive Protein (mg/L)	4.7 (6.6)	4.9 (6.5)	4.5 (6.7)	4.9 (5.9)	5.1 (5.5)	4.6 (6.3)	0.105	0.014	0.524	0.019
**Kidney profile**	Sodium (mmol/L)	140 (2.3)	139.9 (2.3)	140.1 (2.2)	140.1 (2.2)	140.1 (2.2)	140.1 (2.1)	0.199	0.649	0.462	0.451
	Potassium (mmol/L)	4.3 (0.3)	4.3 (0.3)	4.3 (0.3)	4.3 (0.3)	4.3 (0.3)	4.3 (0.3)	0.010	0.437	0.657	0.107
	Chloride (mmol/L)	101.5 (2.3)	101.6 (2.2)	101.4 (2.3)	101.4 (2.3)	101.4 (2.2)	101.6 (2.4)	0.096	0.179	0.428	0.263
	Bicarbonate (mmol/L)	25.9 (2.4)	25.8 (2.3)	25.9 (2.4)	25.6 (2.4)	25.7 (2.4)	25.6 (2.5)	0.140	0.952	0.011	0.064
	Urea (mmol/L)	4.3 (1.4)	4.1 (1.3)	4.5 (1.4)	4.2 (1.2)	4.1 (1.1)	4.3 (1.2)	0.000	0.004	0.061	≤0.001
	Creatinine (mmol/L)	66.9 (15.4)	64.1 (16.1)	68.9 (14.6)	66.8 (14.8)	65.0 (14.3)	68.9 (15.1)	0.000	≤0.001	0.896	≤0.001
	Calcium (mmol/L)	2.4 (0.1)	2.4 (0.1)	2.4 (0.1)	2.4 (0.1)	2.4 (0.1)	2.4 (0.1)	0.051	0.491	0.507	0.202
	Calcium Corrected (mmol/L)	2.3 (0.1)	2.3 (0.1)	2.3 (0.1)	2.3 (0.1)	2.3 (0.1)	2.3 (0.1)	0.351	0.213	≤0.001	0.002
	Phosphorus (mmol/L)	1.2 (0.1)	1.2 (0.1)	1.2 (0.1)	1.2 (0.2)	1.2 (0.2)	1.2 (0.2)	0.113	0.862	≤0.001	≤0.001
	Uric Acid (umol/L)	282 (76.6)	268.0 (77.9)	291.8 (74.2)	289.6 (76.4)	283.4 (74.7)	296.8 (77.8)	≤0.001	0.009	0.016	≤0.001
	Creatine Kinase	206.8 (602.2)	157.2 (595.6)	244.3 (605.6)	168.5 (639.8)	103.2 (116.3)	257.2 (968.0)	≤0.001	0.007	0.204	≤0.001
	Creatine Kinase-1 (ng/mL)	47.3 (107.3)	46.1 (103.0)	48.1 (111.5)	73.5 (289.1)	113.7 (399.8)	30.9 (63.3)	0.864	0.808	0.459	0.914
	Creatine Kinase-2 (U/L)	208.7 (1216.2)	124.1 (280.4)	263.4 (1542.5)	133.3 (275.2)	119.2 (257.1)	147.9 (292.6)	≤0.001	0.002	0.063	≤0.001
	Magnesium (umol/L)	0.8 (0.1)	0.8 (0.1)	0.8 (0.1)	0.8 (0.1)	0.8 (0.1)	0.8 (0.1)	0.712	0.323	0.111	0.203
	Total Protein (g/L)	74.4 (4)	74.2 (4.0)	74.5 (4.0)	74.8 (3.9)	74.9 (3.9)	74.8 (3.8)	0.313	0.771	0.01	0.038
	Homocysteine (umol/L)	9.6 (3.2)	9.4 (3.2)	9.7 (3.1)	9.5 (3.2)	9.4 (3.2)	9.6 (3.2)	0.027	0.371	0.298	0.152
**Liver function**	Bilirubin Total (umol/L)	9.7 (5.6)	9.0 (5.0)	10.3 (5.8)	8.2 (4.7)	8.0 (4.5)	8.4 (4.9)	≤0.001	0.206	≤0.001	≤0.001
	Albumin (g/L)	44.6 (3.4)	44.4 (3.5)	44.8 (3.4)	44.1 (3.6)	44.2 (3.6)	44.1 (3.6)	0.052	0.621	≤0.001	0.001
	Alkaline Phosphatase (U/L)	65 (18.4)	65.8 (19.3)	64.5 (17.7)	69.9 (21.5)	70.9 (23.0)	68.8 (19.6)	0.538	0.080	≤0.001	≤0.001
	ALT (GPT) (U/L)	20.3 (18.1)	19.1 (18.7)	21.1 (17.6)	23.6 (19.2)	23.5 (20.8)	23.7 (17.1)	≤0.001	0.198	≤0.001	≤0.001
	AST (GOT) (U/L)	20.6 (20.1)	18.5 (9.6)	22.1 (24.8)	19.6 (10.7)	19.2 (10.5)	20.1 (10.9)	≤0.001	0.047	0.922	≤0.001
	GGT (U/L)	16.8 (12)	16.6 (12.3)	17.1 (11.9)	22.8 (25.8)	23.8 (28.3)	21.2 (21.5)	0.459	0.312	0.033	0.118
	GGT-2 (U/L)	20.4 (26.9)	22.2 (39.2)	19.1 (13.5)	25 (24.3)	25.1 (24.0)	25.0 (24.6)	0.230	0.690	≤0.001	≤0.001
**Lipid profile**	HDL Cholesterol (mmol/L)	1.5 (0.4)	1.5 (0.4)	1.5 (0.4)	1.4 (0.4)	1.4 (0.4)	1.4 (0.4)	0.063	0.708	≤0.001	≤0.001
	LDL Cholesterol (mmol/L)	2.7 (0.8)	2.7 (0.8)	2.7 (0.8)	2.7 (0.8)	2.7 (0.8)	2.7 (0.8)	0.372	0.290	0.47	0.493
	Triglyceride (mmol/L)	0.9 (0.4)	0.9 (0.5)	0.9 (0.4)	1.2 (0.7)	1.2 (0.7)	1.1 (0.6)	0.283	0.307	≤0.001	≤0.001
**Hormones**	Testosterone Total (nmol/L)	12.1 (12.1)	9.7 (11.3)	13.7 (12.4)	10 (9.5)	9.4 (9.3)	10.7 (9.7)	0.000	0.103	≤0.001	≤0.001
	Estradiol (pmol/L)	292.8 (638.1)	332.8 (895.3)	264.7 (359.2)	262.5 (314.9)	271.5 (332.6)	252.1 (292.9)	0.002	0.780	0.155	0.015
	SHBG (nmol/L)	57.4 (48.1)	60.7 (53.6)	54.9 (43.4)	47.8 (46.9)	49.0 (46.5)	46.1 (47.5)	0.086	0.163	≤0.001	≤0.001
	Free Thyroxine (pmol/L)	14.4 (2.5)	14.3 (2.4)	14.5 (2.6)	14.6 (2.8)	14.5 (2.9)	14.7 (2.6)	0.216	0.251	0.525	0.2
	Free Triiodothyronine	4.4 (0.8)	4.3 (0.8)	4.4 (0.8)	4.7 (0.9)	4.6 (1.0)	4.7 (0.9)	0.294	0.189	≤0.001	≤0.001
	TSH mI (U/L)	2.3 (5.3)	2.2 (4.8)	2.3 (5.7)	2.1 (3.3)	2.2 (4.2)	2.0 (1.8)	0.968	0.906	0.696	0.871
**Iron profile**	Iron (umol/L)	16.4 (7.2)	15.8 (7.8)	16.9 (6.8)	14.7 (6.5)	14.5 (6.5)	14.9 (6.5)	0.002	0.428	≤0.001	≤0.001
	TIBC (umol/L)	63.5 (11.7)	64.4 (12.3)	62.9 (11.2)	66 (11.2)	65.7 (11.4)	66.3 (10.9)	0.089	0.259	≤0.001	≤0.001
	UIBC (umol/L)	42.8 (13)	44.2 (13.8)	41.7 (12.2)	47.2 (13.5)	47.9 (14.0)	46.4 (12.7)	0.024	0.268	≤0.001	≤0.001
	Ferritin (μg/L)	68.8 (70)	59.5 (64.8)	75.3 (72.7)	73.6 (86.5)	71.8 (93.2)	75.7 (78.1)	≤ 0.001	0.107	0.83	≤0.001
**Vitamins**	Folate (nmol/L)	20.3 (8.7)	19.8 (8.9)	20.6 (8.6)	19.1 (7.6)	18.9 (8.0)	19.2 (7.2)	0.051	0.270	0.002	0.003
	Dihydroxyvitamin D (ng/mL)	17.2 (10.9)	16.8 (11.5)	17.4 (10.4)	15.4 (8.6)	14.7 (8.1)	16.1 (9.2)	0.052	0.025	≤0.001	≤0.001
	Vitamin B12 (pmol/L)	312.4 (146.6)	309.3 (160.5)	314.6 (136.1)	290.4 (117.4)	286.7 (117.2)	294.7 (117.6)	0.230	0.362	0.003	0.003

ALT: alanine aminotransferase, AST: aspartate aminotransferase, GGT: gamma-glutamyl transferase, HDL: high-density lipoprotein, LDL: low-density lipoprotein, SHBG: sex hormone-binding globulin, TSH: thyroid-stimulating hormone, TIBC: total iron-binding capacity, UIBC: unsaturated iron-binding capacity, neutrophil (×10^3^ μL): absolute neutrophil count, lymphocyte (×10^3^ μL): absolute lymphocyte count.

**Table 2:** Linear regression analysis evaluating the association between physical activity and the metabolic profiles in each HOMA-IR category.

	Insulin Sensitive (HOMA-IR < 1.85)				Insulin Resistant (HOMA-IR > 1.85)			
	Estimate ^#^	SE	*p*-Value	FDR	Estimate ^#^	SE	*p*-Value	FDR
Handgrip out left	0.044	0.015	0.003	0.027	0.073	0.016	≤0.001	≤0.001
Weight	0.004	0.003	0.329	0.540	0.015	0.005	0.001	0.023
Creatine kinase	0.273	0.071	0.000	0.005	0.207	0.075	0.006	0.052
Creatine kinase-2 (μ/L)	0.202	0.051	0.000	0.005	0.153	0.055	0.005	0.052
AST (GOT) (μ/L)	0.069	0.020	0.001	0.012	0.027	0.022	0.208	0.552
GGT-2 (μ/L)	−0.106	0.032	0.001	0.012	−0.040	0.034	0.229	0.585
Folate (nmol/L)	0.086	0.025	0.001	0.012	0.048	0.027	0.080	0.276
Dihydroxyvitamin D (ng/mL)	0.069	0.031	0.028	0.108	0.093	0.034	0.006	0.052
Creatinine (mmol/L)	0.018	0.009	0.039	0.142	0.040	0.009	0.000	0.001
Triglyceride (mmol/L)	−0.074	0.026	0.005	0.041	−0.036	0.028	0.204	0.552
Hematocrit	0.012	0.005	0.007	0.055	−0.001	0.005	0.997	0.997
White Blood Cell (×10^3^ μL)	−0.041	0.017	0.014	0.100	−0.059	0.018	0.001	0.023
Lymphocyte Auto (×10^3^ μL)	−0.040	0.017	0.023	0.100	−0.055	0.019	0.003	0.042

# This analysis incorporated the interaction between HOMA-IR category and physical activity groups, the estimate represents the offset in the intercept from the active individuals to sedentary baseline. SE: standard error; FDR: false discovery rate.

## Data Availability

The datasets used and/or analysed during the current study are available from the corresponding author on reasonable request.

## Supplementary Materials

The following supporting information can be downloaded at: https://www.mdpi.com/article/10.3390/ijerph191912169/s1, Table S1: linear regression analysis assessing the difference in metabolic profiles between physically active and sedentary males in each HOMA-IR category; Table S2: linear regression analysis assessing the difference in metabolic profiles between physically active and sedentary females in each HOMA-IR category. SE: standard error; FDR: false discovery rate.

## References

[1] Dal Canto E., Ceriello A., Rydén L., Ferrini M., Hansen T.B., Schnell O., Standl E., Beulens J.W. (2019). Diabetes as a cardiovascular risk factor: An overview of global trends of macro and micro vascular complications. Eur. J. Prev. Cardiol..

[2] Bener A., Mohammad A.-G., Ismail A.N., Zirie M., Abdullatef W.K., Al-Hamaq A.O. (2010). Gender and Age-Related Differences in Patients with the Metabolic Syndrome in A Highly Endogamous Population. Bosn. J. Basic Med. Sci..

[3] Mabry R.M., Reeves M.M., Eakin E., Owen N. (2010). Gender differences in prevalence of the metabolic syndrome in Gulf Cooperation Council Countries: A systematic review. Diabet. Med..

[4] Elrayess M.A., Rizk N.M., Fadel A.S., Kerkadi A. (2020). Prevalence and Predictors of Insulin Resistance in Non-Obese Healthy Young Females in Qatar. Int. J. Environ. Res. Public Health.

[5] Luc K., Schramm-Luc A., Guzik T.J., Mikolajczyk T.P. (2019). Oxidative stress and inflammatory markers in prediabetes and diabetes. J. Physiol. Pharmacol..

[6] World Health Organization (1995). Physical status: The use and interpretation of anthropometry. Report of a WHO Expert Committee. World Health Organ. Tech. Rep. Ser..

[7] McLaughlin T., Allison G., Abbasi F., Lamendola C., Reaven G. (2004). Prevalence of insulin resistance and associated cardiovascular disease risk factors among normal weight, overweight, and obese individuals. Metabolism.

[8] St-Onge M.P., Janssen I., Heymsfield S.B. (2004). Metabolic syndrome in normal-weight Americans: New definition of the metabolically obese, normal-weight individual. Diabetes Care.

[9] Owei I., Umekwe N., Provo C., Wan J., Dagogo-Jack S. (2017). Insulin-sensitive and insulin-resistant obese and non-obese phenotypes: Role in prediction of incident pre-diabetes in a longitudinal biracial cohort. BMJ Open Diabetes Res. Care.

[10] Al-Khelaifi F., Donati F., Botrè F., Latiff A., Abraham D., Hingorani A., Georgakopoulos C., Suhre K., Yousri N.A., Elrayess M.A. (2019). Metabolic profiling of elite athletes with different cardiovascular demand. Scand. J. Med. Sci. Sports.

[11] Horton E.S. (2009). Effects of Lifestyle Changes to Reduce Risks of Diabetes and Associated Cardiovascular Risks: Results from Large Scale Efficacy Trials. Obesity.

[12] Ruderman N., Chisholm D., Pi-Sunyer X., Schneider S. (1998). The metabolically obese, normal-weight individual revisited. Diabetes.

[13] Stanford K.I., Goodyear L.J. (2014). Exercise and type 2 diabetes: Molecular mechanisms regulating glucose uptake in skeletal muscle. Adv. Physiol. Educ..

[14] Tuomilehto J., Lindström J., Eriksson J.G., Valle T.T., Hämäläinen H., Ilanne-Parikka P., Keinänen-Kiukaanniemi S., Laakso M., Louheranta A., Rastas M. (2001). Prevention of Type 2 Diabetes Mellitus by Changes in Lifestyle among Subjects with Impaired Glucose Tolerance. N. Engl. J. Med..

[15] Blonde L. (2010). Current Antihyperglycemic Treatment Guidelines and Algorithms for Patients with Type 2 Diabetes Mellitus. Am. J. Med..

[16] Mondon C.E., Dolkas C.B., Reaven G.M. (1980). Site of enhanced insulin sensitivity in exercise-trained rats at rest. Am. J. Physiol. Metab..

[17] James D., Kraegen E.W., Chisholm D.J. (1985). Effects of exercise training on in vivo insulin action in individual tissues of the rat. J. Clin. Investig..

[18] Eriksson J., Taimela S., Koivisto V.A. (1997). Exercise and the metabolic syndrome. Diabetologia.

[19] Schneider S.H.S., Vitug A., Ruderman N. (1986). Atherosclerosis and physical activity. Diabetes Metab. Rev..

[20] Holloszy J.O., Schultz J., Kusnierkiewicz J., Hagberg J.M., Ehsani A.A. (2009). Effects of Exercise on Glucose Tolerance and Insulin Resistance. Acta Medica Scand..

[21] Meigs J.B., D’Agostino R.B., Wilson P.W., Cupples L.A., Nathan D.M., Singer D.E. (1997). Risk variable clustering in the insulin resistance syndrome. The Framingham Offspring Study. Diabetes.

[22] Beck E., Esser N., Paquot N., Scheen A.J. (2009). Metabolically obese normal-weight individuals and metabolically healthy, but obese, subjects. Rev. Med. Suisse.

[23] Oliveros E., Somers V.K., Sochor O., Goel K., Lopez-Jimenez F. (2014). The Concept of Normal Weight Obesity. Prog. Cardiovasc. Dis..

[24] Chaabna K., Mamtani R., Abraham A., Maisonneuve P., Lowenfels A.B., Cheema S. (2022). Physical Activity and Its Barriers and Facilitators among University Students in Qatar: A Cross-Sectional Study. Int. J. Environ. Res. Public Health.

[25] Sayegh S., Van Der Walt M., Al-Kuwari M.G. (2016). One-year assessment of physical activity level in adult Qatari females: A pedometer-based longitudinal study. Int. J. Women’s Health.

[26] Griffin A., Roselli T., Clemens S.L. (2020). Trends in Total Physical Activity Time, Walking, and Vigorous Physical Activity Time in Queensland Adults from 2004–2008. J. Phys. Act. Health.

[27] Majed L., Sayegh S., Chrismas B.C.R. (2022). Reference Walking Speeds for Healthy Young Adults in Qatar: Moderating Effect of Obesity and Physical Activity. SAGE Open.

[28] Pfützner A., Schöndorf T., Hanefeld M., Forst T. (2010). High-Sensitivity C-Reactive Protein Predicts Cardiovascular Risk in Diabetic and Nondiabetic Patients: Effects of Insulin-Sensitizing Treatment with Pioglitazone. J. Diabetes Sci. Technol..

[29] Mahdiani A., Kheirandish M., Bonakdaran S. (2019). Correlation between White Blood Cell Count and Insulin Resistance in Type 2 Diabetes. Curr. Diabetes Rev..

[30] Talukdar S., Bandyopadhyay G., Li D., Xu J., McNelis J., Lu M., Li P., Yan Q., Zhu Y., Ofrecio J. (2012). Neutrophils mediate insulin resistance in mice fed a high-fat diet through secreted elastase. Nat. Med..

[31] Lou M., Luo P., Tang R., Peng Y., Yu S., Huang W., He L. (2015). Relationship between neutrophil-lymphocyte ratio and insulin resistance in newly diagnosed type 2 diabetes mellitus patients. BMC Endocr. Disord..

[32] Barbieri M., Ragno E., Benvenuti E., Zito G.A., Corsi A., Ferrucci L., Paolisso G. (2001). New aspects of the insulin resistance syndrome: Impact on haematological parameters. Diabetologia.

[33] Lima W.G., Martins-Santos M.E.S., Chaves V.E. (2015). Uric acid as a modulator of glucose and lipid metabolism. Biochimie.

[34] Yang M., Ni C., Chang B., Jiang Z., Zhu Y., Tang Y., Li Z., Li C., Li B. (2019). Association between serum total bilirubin levels and the risk of type 2 diabetes mellitus. Diabetes Res. Clin. Pract..

[35] Choi S., Yun K., Choi H. (2013). Relationships between serum total bilirubin levels and metabolic syndrome in Korean adults. Nutr. Metab. Cardiovasc. Dis..

[36] Khoei N.S., Wagner K.-H., Sedlmeier A.M., Gunter M.J., Murphy N., Freisling H. (2022). Bilirubin as an indicator of cardiometabolic health: A cross-sectional analysis in the UK Biobank. Cardiovasc. Diabetol..

[37] Son D.-H., Ha H.-S., Lee Y.-J. (2021). Association of Serum Alkaline Phosphatase with the TG/HDL Ratio and TyG Index in Korean Adults. Biomolecules.

[38] Liu C., Shao M., Lu L., Zhao C., Qiu L., Liu Z. (2021). Obesity, insulin resistance and their interaction on liver enzymes. PLoS ONE.

[39] Azarpazhooh M.R., Najafi F., Darbandi M., Kiarasi S., Oduyemi T., Spence J.D. (2021). Triglyceride/High-Density Lipoprotein Cholesterol Ratio: A Clue to Metabolic Syndrome, Insulin Resistance, and Severe Atherosclerosis. Lipids.

[40] Almuraikhy S., Kafienah W., Bashah M., Diboun I., Jaganjac M., Al-Khelaifi F., Abdesselem H., Mazloum N.A., Alsayrafi M., Mohamed-Ali V. (2016). Interleukin-6 induces impairment in human subcutaneous adipogenesis in obesity-associated insulin resistance. Diabetologia.

[41] Al-Sulaiti H., Diboun I., Agha M.V., Mohamed F.F.S., Atkin S., Dömling A.S., Elrayess M.A., Mazloum N.A. (2019). Metabolic signature of obesity-associated insulin resistance and type 2 diabetes. J. Transl. Med..

[42] Al-Sulaiti H., Diboun I., Banu S., Al-Emadi M., Amani P., Harvey T.M., Dömling A.S., Latiff A., Elrayess M.A. (2018). Triglyceride profiling in adipose tissues from obese insulin sensitive, insulin resistant and type 2 diabetes mellitus individuals. J. Transl. Med..

[43] Diboun I., Al-Mansoori L., Al-Jaber H., Albagha O., Elrayess M.A. (2020). Metabolomics of Lean/Overweight Insulin-Resistant Females Reveals Alterations in Steroids and Fatty Acids. J. Clin. Endocrinol. Metab..

[44] Moriyama K., Urata N., Masuda Y., Oda K., Okuno C., Yamada C., Takashimizu S., Kubo A., Kishimoto N., Nishizaki Y. (2021). Usefulness of Triglyceride to High-Density Lipoprotein Ratio and Alanine Aminotransferase for Predicting Insulin Resistance and Metabolic Syndrome in the Japanese Population. Metab. Syndr. Relat. Disord..

[45] Chiang J.-K., Lai N.-S., Chang J.-K., Koo M. (2011). Predicting insulin resistance using the triglyceride-to-high-density lipoprotein cholesterol ratio in Taiwanese adults. Cardiovasc. Diabetol..

[46] Grossmann M., Thomas M., Panagiotopoulos S., Sharpe K., MacIsaac R.J., Clarke S., Zajac J.D., Jerums G. (2008). Low Testosterone Levels Are Common and Associated with Insulin Resistance in Men with Diabetes. J. Clin. Endocrinol. Metab..

[47] Souteiro P., Belo S., Oliveira S.C., Neves J.S., Magalhães D., Pedro J., Bettencourt-Silva R., Costa M.M., Varela A., Queiros J. (2018). Insulin resistance and sex hormone-binding globulin are independently correlated with low free testosterone levels in obese males. Andrologia.

[48] Benites-Zapata V.A., Urrunaga-Pastor D., Torres-Mallma C., Prado-Bravo C., Guarnizo-Poma M., Lazaro-Alcantara H. (2017). Is free triiodothyronine important in the development of insulin resistance in healthy people?. Diabetes Metab. Syndr..

[49] Ferrannini E., Iervasi G., Cobb J., Ndreu R., Nannipieri M. (2017). Insulin resistance and normal thyroid hormone levels: Prospective study and metabolomic analysis. Am. J. Physiol. Metab..

[50] Chen Y., Wan J., Xia H., Li Y., Xu Y., Lin H., Iftikhar H. (2020). Total iron binding capacity (TIBC) is a potential biomarker of left ventricular remodelling for patients with iron deficiency anaemia. BMC Cardiovasc. Disord..

[51] Dutta D., Maisnam I., Shrivastava A., Sinha A., Ghosh S., Mukhopadhyay P., Mukhopadhyay S., Chowdhury S. (2013). Serum vitamin-D predicts insulin resistance in individuals with prediabetes. Indian J. Med. Res..

[52] Wimalawansa S.J. (2018). Associations of vitamin D with insulin resistance, obesity, type 2 diabetes, and metabolic syndrome. J. Steroid Biochem. Mol. Biol..

[53] Adaikalakoteswari A., Vatish M., Alam M.T., Ott S., Kumar S., Saravanan P. (2017). Low Vitamin B12 in Pregnancy Is Associated With Adipose-Derived Circulating miRs Targeting PPARgamma and Insulin Resistance. J. Clin. Endocrinol. Metab..

[54] Cigerli O., Parildar H., Unal A.D., Tarcin O., Kut A., Eroglu H., Guvener N. (2016). Vitamin Deficiency and Insulin Resistance in Nondiabetic Obese Patients. Acta Endocrinol. (Buchar.).

[55] Zhang H., Wang X., Zhang J., Guan Y., Xing Y. (2022). Early supplementation of folate and vitamin B12 improves insulin resistance in intrauterine growth retardation rats. Transl. Pediatr..

[56] Banfi G., Colombini A., Lombardi G., Lubkowska A. (2012). Metabolic markers in sports medicine. Adv. Clin. Chem..

[57] Nealon D.A., Pettit S.M., Henderson A.R. (1981). Activation of human creatine kinase isoenzymes by pH and various sulfhydryl and chelating agents. Clin. Chem..

[58] Takagi Y., Yasuhara T., Gomi K. (2001). Creatine kinase and its isozymes. Rinsho Byori Suppl..

[59] Oliveira C.P., Sanches P.D.L., de Abreu-Silva E.O., Marcadenti A. (2015). Nutrition and Physical Activity in Nonalcoholic Fatty Liver Disease. J. Diabetes Res..

[60] Evans D.L. (1985). Cardiovascular Adaptations to Exercise and Training. Veter-Clin. N. Am. Equine Pract..

[61] Mairbäurl H. (2013). Red blood cells in sports: Effects of exercise and training on oxygen supply by red blood cells. Front. Physiol..

[62] Hu M., Lin W. (2012). Effects of Exercise Training on Red Blood Cell Production: Implications for Anemia. Acta Haematol..

[63] Seibaek M., Vestergaard H., Burchardt H., Sloth C., Torp-Pedersen C., Nielsen S.L., Hildebrandt P., Pedersen O. (2003). Insulin resistance and maximal oxygen uptake. Clin. Cardiol..

[64] Covington J.D., Tam C.S., Pasarica M., Redman L.M. (2016). Higher circulating leukocytes in women with PCOS is reversed by aerobic exercise. Biochimie.

[65] Pundir C., Kumar P., Jaiwal R. (2019). Biosensing methods for determination of creatinine: A review. Biosens. Bioelectron..

[66] Hammouda O., Chtourou H., Chaouachi A., Chahed H., Ferchichi S., Kallel C., Chamari K., Souissi N. (2012). Effect of Short-Term Maximal Exercise on Biochemical Markers of Muscle Damage, Total Antioxidant Status, and Homocysteine Levels in Football Players. Asian J. Sports Med..

[67] Janssen G.M.E., Degenaar C.P., Menheere P.P.C.A., Habets H.M.L., Geurten P. (1989). Plasma Urea, Creatinine, Uric Acid, Albumin, and Total Protein Concentrations Before and After 15-, 25-, and 42-km Contests. Endoscopy.

[68] Kim E.S., Im J.-A., Kim K.C., Park J.H., Suh S.-H., Kang E.S., Kim S.H., Jekal Y., Lee C.W., Yoon Y.-J. (2007). Improved Insulin Sensitivity and Adiponectin Level after Exercise Training in Obese Korean Youth. Obesity.

[69] Marinho S.M.S.D.A., Mafra D., Pelletier S., Hage V., Teuma C., Laville M., Eduardo J.C.C., Fouque D. (2016). In Hemodialysis Patients, Intradialytic Resistance Exercise Improves Osteoblast Function: A Pilot Study. J. Ren. Nutr..

[70] Wilson-Barnes S.L., Hunt J.E.A., Mendis J., Williams E.L., King D., Roberts H., Lanham-New S.A., Manders R.J.F. (2021). The relationship between vitamin D status, intake and exercise performance in UK University-level athletes and healthy inactive controls. PLoS ONE.

[71] Enhörning S., Struck J., Wirfält E., Hedblad B., Morgenthaler N.G., Melander O. (2011). Plasma Copeptin, A Unifying Factor behind the Metabolic Syndrome. J. Clin. Endocrinol. Metab..

[72] Stella A.B., Yardley J., Francescato M.P., Morrison S. (2018). Fluid Intake Habits in Type 1 Diabetes Individuals during Typical Training Bouts. Ann. Nutr. Metab..

[73] Brunkwall L., Ericson U., Nilsson P.M., Enhörning S. (2020). High water intake and low urine osmolality are associated with favorable metabolic profile at a population level: Low vasopressin secretion as a possible explanation. Eur. J. Nutr..

