# Supplementary material for: Comparing the Metabolic Profiles Associated with Fitness Status between Insulin-Sensitive and Insulin-Resistant Non-Obese Individuals
Source: Int J Environ Res Public Health. 2022 Sep 26;19(19):12169. doi: 10.3390/ijerph191912169 (PMC9564877; doi:10.3390/ijerph191912169)
Supplement: Supplementary file 1 [file ijerph-19-12169-s001.zip › ijerph-1881468-supplementary.pdf]

Table S1. Linear regression analysis assessing the difference of metabolic profiles between physically active and sedentary males in each HOMA-IR category.

	Insulin sensitive (HOMA-IR < 1.85)				Insulin resistant (HOMA-IR > 1.85)			
	**Estimate[#]**	**SE**	**p-value**	**FDR**	**Estimate[#]**	**SE**	**p-value**	**FDR**
Handgrip left	0.057	0.020	0.005	**0.047**	0.093	0.021	<0.0001	**0.001**
Waist size	-0.015	0.005	0.002	**0.034**	-0.005	0.005	0.291	0.748
Creatine kinase	0.394	0.128	0.002	**0.034**	0.313	0.129	0.016	0.283
Creatine kinase-2 (u/l)	0.287	0.089	0.001	**0.031**	0.164	0.089	0.067	0.431
GGT-2 (u/l)	-0.177	0.047	<0.001	**0.007**	-0.036	0.048	0.455	0.759
Folate (nmol/L)	0.146	0.037	<0.001	**0.005**	0.066	0.037	0.075	0.431
Triglyceride (mmol/L)	-0.116	0.040	0.004	**0.045**	-0.047	0.041	0.248	0.748
Bilirubin (umol/L)	0.118	0.042	0.005	**0.048**	0.027	0.043	0.527	0.759

SE: Standard Error; FDR: False Discovery Rate

Table S2. Linear regression analysis assessing the difference of metabolic profiles between physically active and sedentary females in each HOMA-IR category. SE: Standard Error; FDR – False Discovery Rate.

	Insulin sensitive (HOMA-IR < 1.85)				Insulin resistant (HOMA-IR > 1.85)			
	**Estimate**	**SE**	**p-value**	**FDR**	**Estimate**	**SE**	**p-value**	**FDR**
Creatinine	0.009	0.012	0.438	0.689	0.049	0.014	<0.001	**0.025**
Creatine Kinase	0.161	0.072	**0.027**	0.322	0.111	0.079	0.160	0.562
Creatine Kinase 2	0.133	0.054	**0.015**	0.322	0.13	0.062	**0.036**	0.241
White blood Cell ($x10^3$ uL)	-0.049	0.023	**0.037**	0.322	-0.066	0.026	**0.012**	0.125
Dihydroxyvitamin D Total (ng/ml)	0.024	0.047	0.597	0.781	0.157	0.053	**0.003**	0.101
Glucose	-0.004	0.008	0.549	0.746	-0.024	0.009	**0.007**	0.101
Handgrip right	0.037	0.020	0.072	0.383	0.053	0.023	**0.022**	0.174
Handgrip left	0.031	0.021	0.137	0.496	0.047	0.024	**0.047**	0.282
